# Association between depression and endometriosis using data from NHANES 2005–2006

**DOI:** 10.1038/s41598-023-46005-2

**Published:** 2023-10-31

**Authors:** Pan-Wei Hu, Xiao-Le Zhang, Xiao-Tong Yan, Cong Qi, Guo-Jing Jiang

**Affiliations:** Department of Obstetrics and Gynecology, Shuguang Hospital Affiliated to Shanghai University of Traditional Chinese Medicine, 528 Zhangheng Road, Shanghai, China

**Keywords:** Diseases, Medical research

## Abstract

Studies on the association between depression and self-reported endometriosis are limited, and further studies are required to investigate this association. Data were collected from the National Health and Nutrition Examination Survey database (2005–2006). Based on the inclusion and exclusion criteria, 100 participants with self-reported endometriosis and 1295 participants without self-reported endometriosis were included, representing a total population of 64,989,430. Depression severity was assessed using the Patient Health Questionnaire 9 (PHQ9). A survey-weighted logistic regression analysis was performed to explore the association between depression and endometriosis. Subgroup analyses were conducted to explore heterogeneity. The prevalence of endometriosis was 7.17%. A significant positive association was found between the PHQ9 score and endometriosis. After adjusting for all covariates, the PHQ9 score positively correlated with endometriosis. Furthermore, compared with the participants without depression, those with moderate depression were more prone to have endometriosis both in unadjusted and fully adjusted model. However, the relationship between severe depression and endometriosis was not significant in all models (*P* > 0.05). Our findings highlight the influence of depression on the prevalence of self-reported endometriosis. Further studies are required to elucidate the causal relationship between depression and self-reported endometriosis.

## Introduction

Endometriosis is a complex chronic inflammatory disease characterized by the appearance of endometrial tissues (glands and stroma) outside the uterus^1, 2^. As a common benign gynecological disorder, the incidence of endometriosis is thought to reach 10% in females of childbearing age; however, the incidence may be even higher owing to the high rate of misdiagnosis and missed diagnosis^3, 4^. Although numerous studies have been conducted, the etiology of endometriosis remains unclear^5^. Clinically, endometriosis is closely associated with pelvic pain, menstrual cramps, and infertility, imposing enormous financial and social burdens on individuals, families, and society^6^. Further research is required to identify better options for preventing and managing endometriosis.

Depression is a common psychiatric disorder adversely affecting psychological and physical health worldwide, making it a substantial public health issue^7^. Clinically, depression is associated with many chronic diseases, and patients with depression are more prone to worsening disease progression than patients without depression^8, 9^. Studies have found a strong correlation between depression and gynecological diseases, including infertility and cancer^10^. Depression may also be an important risk factor for sexual dysfunction^11^. Given these perspectives, it is worth exploring whether depression is closely associated with the risk of endometriosis. However, no studies have explored the link between depression and the prevalence of endometriosis after adjusting for multiple covariates such as age, race, and other exposures.

This study explores the association between depression and self-reported endometriosis using data from the National Health and Nutrition Examination Survey (NHANES) (2005–2006), elucidating the role of depression in endometriosis prevalence.

## Methods

### Study design and participants

**Figure 1:**
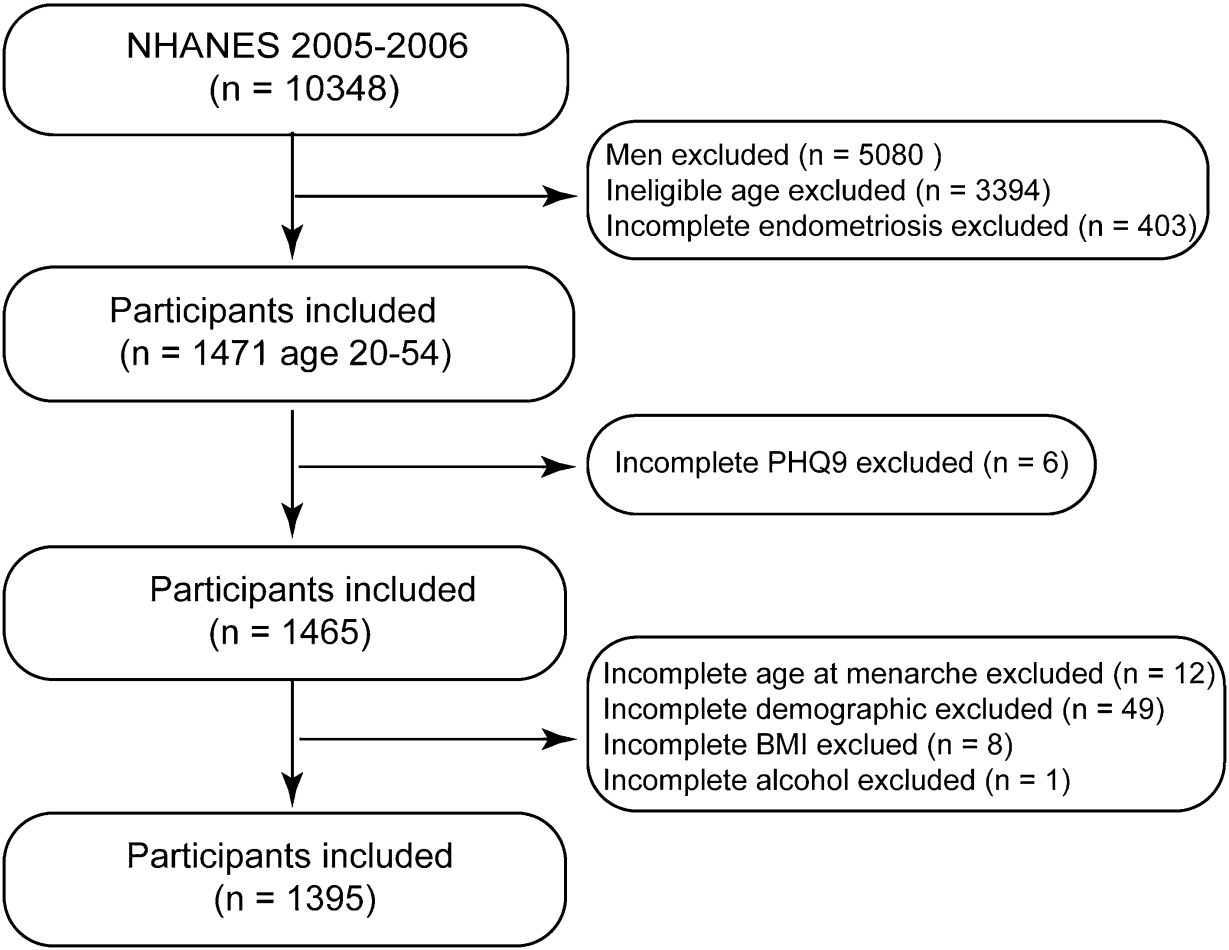
Flow chart of the screening process from National Health and Nutrition Examination Survey (2005–2006). BMI, body mass index.

Since 1999, the NHANES database has provided a continuous and multistage probability sample for assessing health and nutritional status in the United States^12^. Survey participants were interviewed at home. Subsequently, physical and laboratory examinations were performed at a mobile examination center (MEC). The National Center for Health Statistics Research Ethics Review Board provided ethics approval (Protocol #2005-06) for all potential study protocols in the NHANES, and written informed consent was obtained from all participants. Therefore, no external ethical approval and informed consent were required. All analyses in this study were performed in accordance with the NHANES guidelines and regulations. After a comprehensive search and screening of the NHANES database, participants from 2005 to 2006 were included in this study. Male participants were excluded, and only females aged 20–54 years were included. Simultaneously, individuals with incomplete information on self-reported endometriosis, Patient Health Questionnaire 9 (PHQ9), and covariates were excluded. The flowchart of the screening process is shown in Fig. 1.

### Exposure and outcome definitions

PHQ9 is a reliable questionnaire for rating mood based on standard diagnostic criteria for depression^13, 14^. The PHQ9 scale consists of nine items rated from 0 to 3, with the total score ranging from 0 to 27^15^. The depression severity of the survey participants was evaluated using PHQ9, administered in an MEC^16^. The depression was defined as a total PHQ9 score ≥ 10^17^. For participants with depression, the degree of depression was further divided into moderate (total PHQ9 score: 10–14) and severe depression (total PHQ9 score ≥ 15)^18^. The grade of depression and PHQ9 score of each participant were then calculated for further analysis.

Self-reported endometriosis was diagnosed based on the “rhq360” questionnaire administered in the MEC. The structured questionnaire comprised the question, “Has a doctor or other health professional ever told you that you have endometriosis?”^19^. Individuals who answered “yes” were categorized into the case group with self-reported endometriosis, and individuals who answered “no” were classified into the control group without self-reported endometriosis.

### Potential covariates

Demographic covariates, including age, race, marital status, educational level, and poverty level, were obtained through home interviews. According to the age distribution, the participants were divided into three groups: first (20–29 years), second (30–40 years), and third (41–54 years) tertiles. Marital status was defined as married, divorced, separated, or spinster^20^. The participants’ educational level was recorded as less than high school, high school, or college and above^21^. Poverty level was evaluated based on the poverty income ratio (PIR) and classified as follows: low-income (PIR < 1.35), medium-income (1.35 ≤ PIR < 3.0), and high-income (PIR ≥ 3.0)^22^. Health-related covariates, including smoking status and alcohol use, were evaluated in the MEC. According to the smoking and tobacco use questionnaire, smoking status was categorized as never, former, or current, whereas alcohol consumption was classified as never, former, mild, moderate, or heavy. Age at menarche and history of pregnancy were included as disease-related covariates using “rhq” 010 and 031 questionnaires^23, 24^. Body mass index (BMI) was classified as underweight (BMI < 18.5 kg/m^2^), normal weight (BMI = 18.5–24.9 kg/m^2^), overweight (BMI = 25–29.9 kg/m^2^), or obese (BMI ≥ 30 kg/m^2^)^25^.

### Statistical analysis

Due to the complex multistage sampling design of the NHANES, all data were merged and weighted using wtmec2yr under the NHANES protocol^26^. The baseline characteristics of the participants were first compared using Student’s *t*-test for continuous variables and the chi-square test for categorical variables^27^. For descriptive statistics, the variables were expressed as weighted means (standard error). Categorical variables were presented as numbers (weighted percentages). Next, weighted univariate and multivariate logistic regression models were used to evaluate the correlation between depression and endometriosis. In the multivariate logistic regression analysis, three models were constructed: (1) Model 1 (no covariate were adjusted), (2) Model 2 (adjusted for variables found to be significant in the univariate logistic regression analysis), and (3) Model 3 (adjusted for all covariates). Finally, the statistical *P* for interactions between the covariates and the PHQ9 score was calculated, and subgroup analyses were conducted to further clarify these results. The weighted logistic regression and subgroup analysis results are expressed as odds ratios (OR) and 95% confidence intervals (CI). All analyses were performed using R and R studio (version 4.2.1), and *P* < 0.05 was used to indicate statistical significance.

## Results

### Population characteristics

Descriptive characteristics of the study participants are presented in Table 1. After a series of screenings, 1395 participants were included from the NHANES database (2005–2006), representing a total population sample of 64,989,430. Of these, 100 (7.17%) individuals had self-reported endometriosis, whereas 1295 (92.83%) did not. Significant differences in age, race, PIR, and the PHQ9 score were found between patients with and without self-reported endometriosis (*P* < 0.05).

**Table 1:** Characteristics of the participants in the NHANES (2005–2006).

Variables	Total (n = 1395)	Control group (n = 1295)	Self-reported endometriosis case group (n = 100)	*P*-value
Age (years)	37.52 ± 0.22	37.12 ± 0.24	41.31 ± 0.67	< 0.001
Age at menarche (years)	12.58 ± 0.07	12.60 ± 0.07	12.40 ± 0.24	0.411
Alcohol				0.361
Never	256 (18.35%)	246 (13.71%)	10 (11.72%)	
Former	217 (15.56%)	201 (12.77%)	16 (15.11%)	
Mild	337 (24.16%)	304 (26.62%)	33 (32.25%)	
Moderate	276 (19.78%)	250 (21.93%)	26 (25.83%)	
Heavy	309 (22.15%)	294 (24.96%)	15 (15.09%)	
BMI	28.41 ± 0.40	28.39 ± 0.42	28.62 ± 1.02	0.83
Education				0.207
Less than high school	287 (20.57%)	278 (13.08%)	9 (8.21%)	
High school	292 (20.93%)	271 (21.09%)	21 (28.08%)	
College or above	816 (58.49%)	746 (65.82%)	70 (63.71%)	
Marital				0.348
Married	932 (66.81%)	868 (67.91%)	64 (64.09%)	
Divorced/separated	188 (13.48%)	169 (14.26%)	19 (20.26%)	
Spinsterhood	275 (19.71%)	258 (17.83%)	17 (15.65%)	
Poverty ratio income	3.16 ± 0.06	3.12 ± 0.06	3.59 ± 0.18	0.031
Pregnant history				0.889
Yes	1178 (84.44%)	1097 (79.52%)	81 (80.28%)	
No	217 (15.56%)	198 (20.48%)	19 (19.72%)	
Race				0.013
Non-Hispanic White	644 (46.16%)	583 (68.12%)	61 (79.66%)	
Non-Hispanic Black	320 (22.94%)	292 (12.67%)	28 (12.02%)	
Mexican American	302 (21.65%)	298 (8.72%)	4 (1.56%)	
Other	129 (9.25%)	122 (10.49%)	7 (6.75%)	
Smoke				0.171
Never	883 (63.3%)	824 (59.07%)	59 (52.75%)	
Former	239 (17.13%)	222 (18.64%)	17 (16.40%)	
Current	273 (19.57%)	249 (22.30%)	24 (30.85%)	
Depression				0.185
No	1285 (92.11%)	1200 (93.61%)	85 (89.51%)	
Moderate	72 (5.16%)	59 (4.12%)	13 (8.51%)	
Severe	38 (2.72%)	36 (2.27%)	2 (1.97%)	
PHQ9 score	3.02 (0.17)	2.93 (0.16)	3.87 (0.49)	0.049

Mean ± SE for continuous variables: *P* value was calculated by logistic regression analysis. % for categorical variables: P value was calculated by chi-square test. Classification of depression: no (total PHQ9 score < 10), moderate depression (total PHQ9 score 10–14), severe depression (total PHQ9 score ≥ 15). BMI, body mass index; PHQ, patient health questionnaire.

### Weighted logistic regression

To explore the link between depression and self-reported endometriosis, weighted univariate and multivariate logistic regression analyses were performed. As shown in Table 2, Model 1 was unadjusted; Model 2 was adjusted for age, PIR, and race; and Model 3 was adjusted for all covariates. Compared with the participants without depression, the association between the PHQ9 score and endometriosis was robust and significant in Model 1 (OR = 1.05, 95% CI 1.01–1.10), Model 2 (OR = 1.07, 95% CI 1.02–1.12), and Model 3 (OR = 1.06, 95% CI 1.01–1.11). The same relationship was verified between moderate depression and endometriosis through Model 1 (OR = 2.16, 95% CI 1.02–4.57), Model 2 (OR = 2.30, 95% CI 1.11–4.75), and Model 3 (OR = 2.49, 95% CI 1.09–5.69). However, the relationship between severe depression and endometriosis was not significant in any model (*P* > 0.05). Compared with the age group of 20–29 years, the age group of 41–54 years was found to be positively correlated with endometriosis prevalence in Model 1 (OR = 3.33, 95% CI 1.45–7.63), Model 2 (OR = 2.88, 95% CI 1.05–7.93), and Model 3 (OR = 2.91, 95% CI 1.01–8.41). With regards to race, compared with non-Hispanic White, Mexican American race was negatively correlated with endometriosis prevalence in Model 1 (OR = 0.15, 95% CI 0.05–0.47), Model 2 (OR = 0.19, 95% CI 0.05–0.65), and Model 3 (OR = 0.21, 95% CI 0.07–0.70). Regarding the PIR covariate, no significant relationship was identified between medium- or high-income and endometriosis across all models compared with low-income (*P* > 0.05).

**Table 2:** Association between the covariates and odds of endometriosis.

	Model 1, *OR* (95% *CI*)	Model 2, *OR* (95% *CI*)	Model 3, *OR* (95% *CI*)
PHQ9 score	1.05 (1.01, 1.10)	1.07 (1.02, 1.12)	1.06 (1.01, 1.11)
Depression			
No	Reference	Reference	Reference
Moderate	2.16 (1.02, 4.57)	2.30 (1.11, 4.75)	2.49 (1.09, 5.69)
Severe	0.91 (0.13, 6.53)	1.19 (0.11, 13.07)	0.97 (0.13, 7.37)
Age (years)			
20–29	Reference	Reference	Reference
30–40	2.12 (0.86, 5.20)	2.04 (0.69, 6.04)	2.26 (0.77, 6.62)
41–54	3.33 (1.45, 7.63)	2.88 (1.05, 7.93)	2.91 (1.01, 8.41)
Race			
Non-Hispanic White	Reference	Reference	Reference
Non-Hispanic Black	0.81 (0.46, 1.45)	0.86 (0.46, 1.63)	0.81 (0.40, 1.65)
Mexican American	0.15 (0.05, 0.47)	0.19 (0.05, 0.65)	0.21 (0.07, 0.70)
Other	0.55 (0.24, 1.26)	0.66 (0.27, 1.62)	0.65 (0.29, 1.45)
Poverty ratio income			
Low	Reference	Reference	Reference
Medium	0.75 (0.27, 2.12)	0.71 (0.24, 2.05)	0.84 (0.35, 2.04)
High	1.52 (0.65, 3.58)	1.23 (0.45, 3.36)	1.65 (0.59, 4.57)

Model 1: No covariate were adjusted. Model 2: Adjusted for age, race, and poverty level but not for the covariate itself. Model 3: Adjusted for age, age at menarche, alcohol use, BMI, education level, marital status, poverty level, history of pregnancy, race, and smoking status. *P* value was calculated by logistic regression analysis. Classification of depression: no (total PHQ9 score < 10), moderate depression (total PHQ9 score 10–14), severe depression (total PHQ9 score ≥ 15). BMI, body mass index; PHQ, patient health questionnaire.

### Subgroup analyses

**Figure 2:**
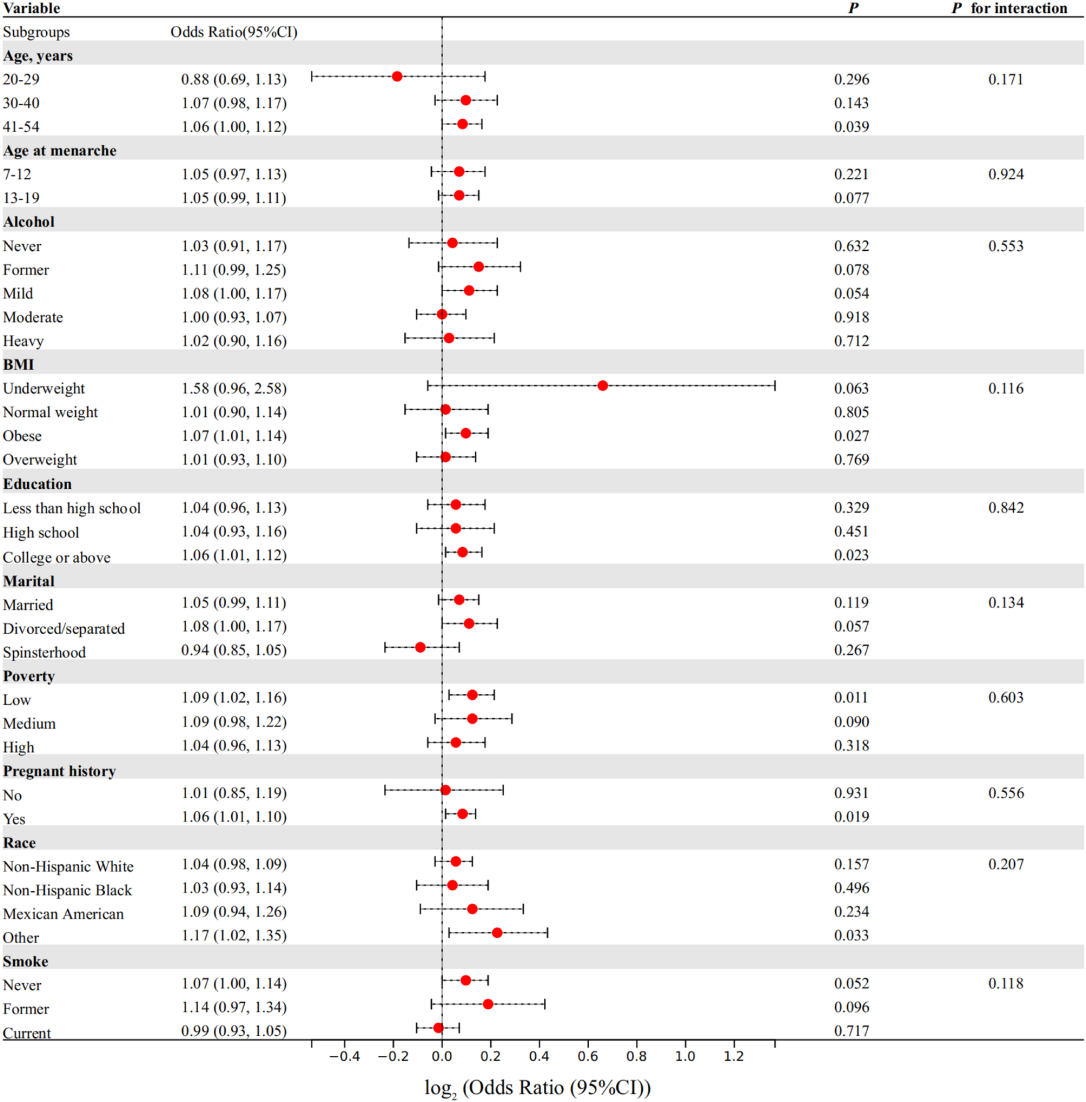
Subgroup analyses on the effect of interaction between the covariates and Patient Health Questionnaire 9 score on the prevalence of endometriosis. *P* value was calculated by *P* for interaction and logistic regression analysis. BMI, body mass index. PIR, poverty income ratio.

To assess the influence of potential effect modifiers on the prevalence of self-reported endometriosis, *P* for interaction and subgroup analyses were conducted (Fig. 2). The results showed no significant interactions between the covariates and PHQ9 score (*P* for interaction > 0.05). The ORs in all subgroups were greater than one, indicating a robust positive relationship between depression and the prevalence of endometriosis. Although the OR was lesser than one in the subgroups with age 20–29 years (OR = 0.88, 95% CI 0.69–1.13, *P* = 0.296), spinster status (OR = 0.94, 95% CI 0.85–1.05, *P* = 0.267), and current smoking status (OR = 0.99, 95% CI 0.93–1.05, *P* = 0.717), the *P* value was not significant.

## Discussion

To explore the relationship between depression and endometriosis, we conducted a cross-sectional study of 1395 NHANES participants, of whom 7.17% had self-reported endometriosis. Multivariate logistic regression analysis was used to assess the relationship between depression and self-reported endometriosis through independent evaluations. Here, we report that the PHQ9 scores positively correlate with endometriosis prevalence. Particularly, participants with moderate depression exhibited a significantly association with the prevalence of endometriosis compared with those without depression. Our results were relatively robust after adjusting for related covariates. Our findings highlight the role of depression in endometriosis prevention.

Although surgical and drug interventions are currently available for the management of endometriosis, these therapies remain insufficient owing to the high rate of recurrence^28–30^. Depression is strongly associated with gynecological diseases, including endometriosis^10^. Participants with moderate depression may be prone to self-reported endometriosis, consistent with pathogenic role of PHQ9 reported previously^18^. In certain situations, depression can contribute to disease progression. For example, severe depressive symptoms are closely associated with an elevated risk of cardiovascular disease^31, 32^. Notably, women are more likely to experience depression than men^33, 34^. In patients with chronic pelvic pain, higher depression scores were observed in the physical, psychological, social, and environmental domains, likely reflecting the negative effects of depression on quality of life^35^. A cross-sectional study showed that depression may postpone menopause by targeting specific biological mechanisms^36^. Our findings highlighted the influence of depression on the prevalence of self-reported endometriosis.

In this study, age and race were identified as independent risk factors for endometriosis. Population-based studies in China suggest that endometriosis is most common in women aged 15–54 years, with the maximum risk found in the 15–24 years age range; the prevalence of endometriosis subsequently decreases continuously with age^37, 38^. However, in the United States, the highest incidence of endometriosis is observed in women aged 36–45 years^39^. In the present study, participants aged 41–54 years had a higher prevalence of endometriosis than those in the youngest age group (20–29 years). This may be explained by the chronic-continuous morbidity pattern consistent with endometriosis. Furthermore, we found that Mexican–American participants were less vulnerable to endometriosis than White participants. Overall, White patients have a higher chance of endometriosis diagnosis than non-White patients^40^. However, due to access to good medical conditions, White patients had a better prognosis, accompanied by lower mortality and cost compared with patients of other races^41^.

This study had certain limitations. First, this was a cross-sectional study; thus, causality could not be determined. Therefore, it is worth investigating whether depression and self-reported endometriosis mutually influence each other. Second, due to the characteristic features of PHQ9 and endometriosis, only participants in a 1-year cycle (2005–2006) were enrolled in this study, which may have led to selection bias. Third, given the high loss rate in covariate “diagnosis age of endometriosis,” the results may be subject to a certain margin of bias. Finally, sampling errors inherent in the NHANES data cannot be ruled out. Considering these limitations, large prospective cohort studies are required to confirm our results.

In conclusion, we found a strong positive association between the PHQ9 score and self-reported endometriosis. Moderate depression significantly and positively correlated with the prevalence of self-reported endometriosis. Our study sheds light on the risk of depression in patients with endometriosis. Further studies are required to elucidate a causal relationship between depression and self-reported endometriosis.

## Data Availability

The datasets used in this study are freely available from the National Health and Nutrition Examination Survey (NHANES). The data can be accessed at https://www.cdc.gov/nchs/nhanes.

## Abbreviations

PHQ9: Patient Health Questionnaire 9
MEC: Mobile examination center
NHANES: National Health and Nutrition Examination Survey
PIR: Poverty income ratio
BMI: Body mass index
